# 2831. Efficacy and Safety of Gepotidacin for Uncomplicated Urinary Tract Infection: Pooled Subgroup Analyses of the EAGLE-2 and EAGLE-3 Randomized Phase 3 Trials

**DOI:** 10.1093/ofid/ofad500.2441

**Published:** 2023-11-27

**Authors:** Thomas M Hooton, Caroline R Perry, Salim Janmohamed, Amanda Sheets, Jeremy Dennison, Helen Millns, Emily Jarvis, Chun Huang

**Affiliations:** University of Miami, Miami, Florida, USA, Coral Gables, FL; GSK, Collegeville, PA, USA, Collegeville, Pennsylvania; GSK, Brentford, UK, Brentford, England, United Kingdom; GSK, Collegeville, PA, USA, Collegeville, Pennsylvania; GSK, Brentford, UK, Brentford, England, United Kingdom; GSK, Stevenage, UK, Stevenage, England, United Kingdom; GSK, Stevenage, UK, Stevenage, England, United Kingdom; GSK, Collegeville, PA, USA, Collegeville, Pennsylvania

## Abstract

**Background:**

Two large Phase 3 trials showed that gepotidacin, a first-in-class oral triazaacenaphthylene bactericidal antibiotic, was efficacious in treating uncomplicated urinary tract infection (uUTI) in females and had an acceptable safety profile consistent with prior gepotidacin trials. We now present a pooled analysis of the EAGLE-2 and EAGLE-3 trials to evaluate gepotidacin efficacy and safety in patient subgroups.

**Methods:**

Data were pooled from EAGLE-2 and EAGLE-3, near identical global, Phase 3, randomized, double-blind, double-dummy, active-controlled non-inferiority trials comparing oral gepotidacin (1500mg) to nitrofurantoin (100mg), both twice daily for 5 days. Females aged ≥ 12 years with ≥ 2 uUTI symptoms were eligible. Therapeutic success (combined clinical and microbiological success) was determined at the test-of-cure visit (Day 10–13) for patient subgroups – age, geographic region, history of recurrent uUTI, diabetes, and renal function. Efficacy subgroup analyses were conducted in the pooled microbiological intent-to-treat population of patients with baseline uropathogens (≥ 10^5^ CFU/mL) susceptible to nitrofurantoin (micro-ITT NTF-S) and were adjusted for study. Pooled adverse event (AE) data for the overall population are presented.

**Results:**

Both studies stopped early for efficacy (per independent data monitoring committee review). Among 1201 patients in the pooled micro-ITT NTF-S population, mean age was 51.5 years; 54% were aged > 50 years (**Table 1**). Across subgroups, differences in therapeutic success numerically favored gepotidacin vs nitrofurantoin (**Table 2**). Treatment differences were higher for > 50 years vs other age groups and for mild renal impairment vs normal renal function, and were similar within other subgroups. AEs were reported in 35% (gepotidacin) and 23% (nitrofurantoin) of patients (**Table 3**); most were mild to moderate and gastrointestinal in nature. There were few serious AEs.

**Figure:**
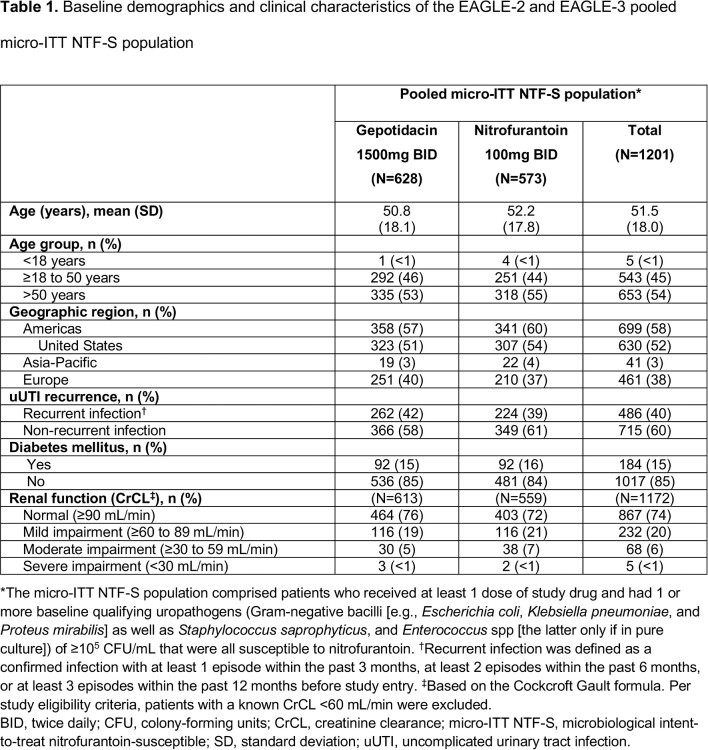

**Figure:**
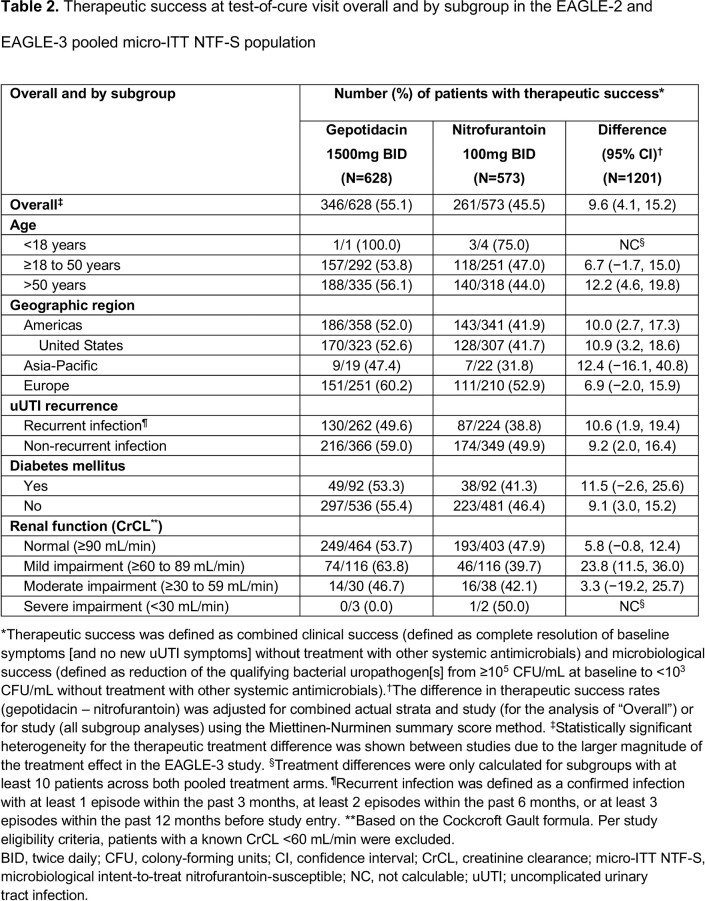

**Figure:**
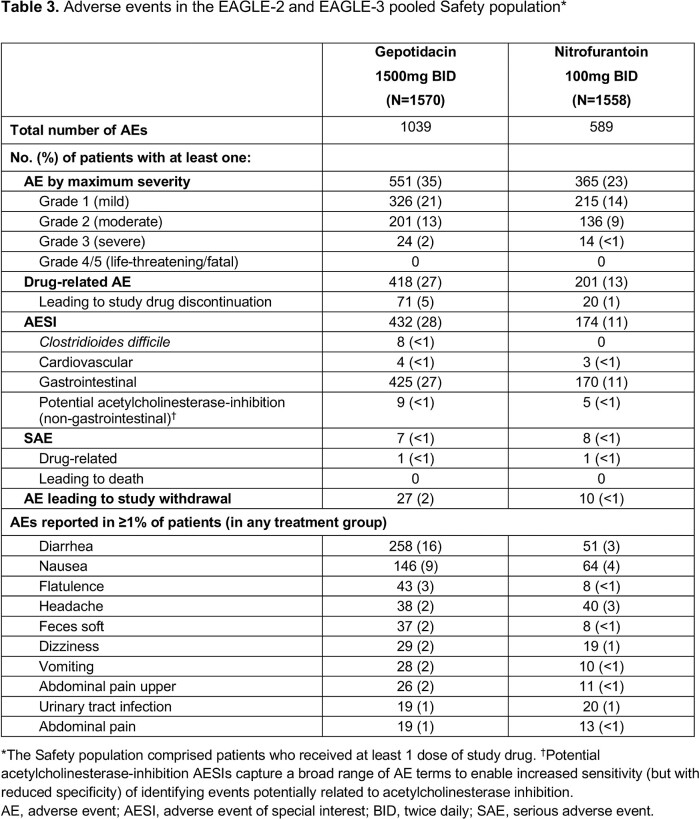

**Conclusion:**

Gepotidacin generally demonstrated consistent therapeutic efficacy in patient subgroups, including patients aged > 50 years and those with a history of recurrent uUTI (subgroups at higher risk of treatment failure). There were no new or concerning safety signals. Gepotidacin has potential as a novel oral treatment for uUTI in key patient subgroups.

**Disclosures:**

**Thomas M. Hooton, MD**, GSK: Advisor/Consultant **Caroline R. Perry, PhD**, GSK: Employee and shareholder **Salim Janmohamed, MD**, GSK: Employee and shareholder **Amanda Sheets, PhD**, GSK: Employee and shareholder **Jeremy Dennison, MD**, GSK: Employee and shareholder **Helen Millns, PhD**, GSK: Employee and shareholder **Emily Jarvis, MSc**, GSK: Employee and shareholder **Chun Huang, PhD**, GSK: Employee and shareholder

